# Dose-de-escalated focal radiotherapy for primary cutaneous T-cell lymphoma: a lesion-based retrospective cohort study

**DOI:** 10.2340/1651-226X.2026.45728

**Published:** 2026-05-25

**Authors:** Philipp Linde, Kathrin Wagner, Johannes Rosenbrock, Michael Oertel, Simone Ferdinandus, Jiaqi Fan, Emmanouil Fokas, Christian Baues

**Affiliations:** a Department of Radiation Oncology, Cyberknife and Radiation Therapy, Faculty of Medicine and University Hospital of Cologne, University of Cologne, Cologne, Germany; b Center for Integrated Oncology (CIO), University Hospital of Cologne, Faculty of Medicine and University of Cologne, Cologne, Germany; c Department of Radiation Oncology, University Hospital Muenster, Muenster, Germany; d Department of Radiation Oncology, Ruhr University Bochum, Bochum, Germany; e Medical Faculty, Marienhospital Herne, Herne, Germany

**Keywords:** cutaneous T-cell lymphoma, mycosis fungoides, radiotherapy, dose hypofractionation, local control, treatment outcome, patient reported outcome measures

## Abstract

**Background and purpose:**

Radiotherapy (RT) is effective for cutaneous T-cell lymphoma (CTCL), but the optimal focal dose remains debated. We evaluated lesion response, local control, toxicity and patient satisfaction after 2 × 4 Gy compared with higher-dose regimens.

**Patient/material and methods:**

This single-centre retrospective study included CTCL patients treated with RT between 1990 and 2019 at a German university hospital. Lesions receiving 2 × 4 Gy (low-dose) were compared with lesions treated with higher doses (median 34 Gy). Endpoints were complete response (CR), overall response (OR), 1- and 5-year local control rate (LCR; Kaplan–Meier/log-rank), in-field relapse and toxicity (common terminology criteria for adverse events/ Radiation Therapy Oncology Group (RTOG)-European Organisation for Research and Treatment of Cancer). Patient satisfaction Freiburg Index of Patient Satisfaction (FIPS) was assessed in a subsample.

**Results:**

Thirty-two patients with 227 lesions were analysed (195 low-dose; 32 higher-dose). CR was achieved in 88.2% vs 90.6% and OR in 94.4% vs 100%, respectively (low- vs higher-dose). LCR was inferior after low-dose RT, with 1- and 5-year LCR estimates of 82 and 67% versus 100 and 96% after higher-dose RT (log-rank *p* = 0.013). In-field relapse occurred in 20.1% (37/184) vs 3.6% (1/28) of lesions with follow-up (Fisher *p* = 0.034). No toxicity > grade 2 was observed, and did not differ between groups (*p* = 0.695 and *p* = 0.657). In 11 FIPS questionnaires, satisfaction was high (mean score 1.27; median 1).

**Interpretation:**

Low-dose 2 × 4 Gy yields excellent initial response with minimal toxicity, but inferior local control compared with higher-dose focal RT. Findings support intent-based dosing: 2 × 4 Gy for symptom-driven treatment with re-irradiation as a pragmatic option, and higher-dose RT when durable local control is the primary objective.

## Introduction

Cutaneous T-cell lymphomas (CTCLs) are rare malignancies primarily involving the skin, with mycosis fungoides as the most frequent subtype [1, 2]. Disease course is heterogeneous, and treatment is often multimodal, ranging from skin-directed therapies to systemic approaches depending on stage, symptoms and disease burden [3, 4].

Radiotherapy (RT) is a key treatment option in CTCL because of the pronounced radiosensitivity of cutaneous lymphoma infiltrates [1, 5]. Both total skin electron beam therapy (TSEBT) and focal/local RT are established components of care. In routine practice, focal RT is frequently chosen for symptomatic, therapy-refractory or cosmetically relevant lesions, allowing field-limited treatment with flexible target shaping and a generally favourable toxicity profile [1, 5].

Despite consistently high response rates after RT, relapses are common in CTCL, and the optimal dose for focal treatment remains debated [5]. Higher-dose focal RT can provide durable control, but requires multiple fractions and may constitute a relevant logistic burden, particularly in frail patients or in symptom-driven palliative scenarios [3–5].

This has fuelled interest in ultra-hypofractionated regimens aiming to preserve excellent response while reducing patient burden and facilitating re-irradiation if needed.

Prior studies have reported very high response rates after low-dose focal RT in CTCL with favourable tolerability; however, dose concepts and endpoints vary, and the relationship between rapid lesion response and durability of in-field control remains clinically relevant [6, 7]. In the palliative setting, Neelis et al. reported excellent responses using 8 Gy delivered in two fractions (2 × 4 Gy) and highlighted the practicality of re-irradiation for relapsing lesions [6]. Recent European Organisation for Research and Treatment of Cancer (EORTC) expert recommendations acknowledge the increasing use of low-dose RT in CTCL, but emphasise the lack of controlled trials defining optimal doses across entities, leaving clinicians to individualise treatment [5, 8]. In this context, lesion-based real-world comparisons of commonly used regimens can inform intent-based dose selection, while not replacing the need for prospective subtype-specific studies.

Larger lesion-based cohorts have since revisited dose de-escalation and suggest that while initial responses remain high at low doses, durability of local control may improve with moderate dose escalation, underscoring the trade-off between convenience and long-term control [7, 9, 10].

Against this background, the aim of this study was to evaluate focal low-dose RT (2 × 4 Gy) as a pragmatic short-course approach in CTCL and to compare it with higher-dose focal RT in a lesion-based retrospective cohort. We assessed initial response (complete and overall response [OR]), durability of local control, in-field relapse and toxicity. In addition, patient satisfaction was explored using the Freiburg Index of Patient Satisfaction (FIPS) in a subsample.

## Patients/material and methods

This was a single-centre, retrospective, lesion-based cohort study of focal RT for CTCL lesions treated at a German university hospital between January 1990 and December 2019.

### Eligibility criteria

Patients were eligible if they had a confirmed diagnosis of a primary cutaneous lymphoma and received focal/local RT to cutaneous lesions [11]. Diagnostic confirmation was established in routine clinical care, including histopathology when available and stage-appropriate imaging as documented. RT had to correspond either to the low-dose regimen of 8 Gy in two fractions (2 × 4 Gy) or to a higher-dose focal RT schedule. Patients with cutaneous B-cell lymphoma (CBCL) were excluded. In addition, cases treated with fractionation schemes deviating from the predefined regimens (e.g. 3 × 4 Gy or 2 × 2 Gy) were excluded. In patients with Sézary syndrome (SS), focal RT was administered for symptom-driven control of selected cutaneous lesions rather than as systemic disease-directed therapy.

### Data sources and study workflow

Patient charts were reviewed and filtered according to predefined eligibility criteria. Variables were extracted at patient and lesion level, including diagnosis category (mycosis fungoides, SS, other CTCL), lesion location and size, RT parameters (dose/fractionation, beam type/energy, bolus/flab), and documented systemic/local therapies and TSEBT exposure. Tumour/nodus/metastasis (TNM) status prior to irradiation was recorded when available; blood involvement (‘B’) was not analysed due to insufficient documentation. Missing chart information was coded as ‘not documented’. ‘Other CTCL’ was analysed as a pooled category because individual non-MF/SS CTCL entities were too infrequent for meaningful subtype-specific analyses, and more granular classification was not consistently available in routine documentation over the long inclusion period.

### Unit of analysis and treatment groups

The unit of analysis was the irradiated lesion. Lesions were grouped according to delivered dose concept: (1) low-dose RT of 8 Gy in two fractions (2 × 4 Gy) and (2) higher-dose focal RT. Dose concept was chosen clinically and evolved over time, with higher-dose RT predominating earlier and 2 × 4 Gy adopted later. Patients may have multiple lesions, and some patients received both dose concepts over time.

### RT technique

Target definition for superficial electron fields was based on the clinically visible lesion with an additional clinical field margin according to institutional practice; formal clinical target volume (CTV)/ planning target volume (PTV) contouring was not routinely applied for these superficial fields. Beam type/energy and bolus/flab use were recorded at the lesion level. In the higher-dose group, fractionation was in the vast majority 2 Gy per fraction (Table 1), making equivalent dose in 2-Gy fractions (EQD2) effectively equivalent to the physical total dose. The 2 × 4 Gy regimen was typically delivered on two consecutive days, depending on scheduling logistics.

**Table 1:** Lesion and treatment characteristics by dose group (lesion-based cohort).

Characteristic	Low-dose RT 2 × 4 Gy (*n* = 195 lesions)	Higher-dose RT (*n* = 32 lesions)	Total (*n* = 227 lesions)
**CTCL diagnosis (lesion-level)**			
Mycosis fungoides	148 (76%)	31 (97%)	179 (79%)
Sézary syndrome	8 (4%)	1 (3%)	9 (4%)
Other CTCL	39 (20%)	0 (0%)	39 (17%)
**Lesion location**			
Trunk	85 (43.6%)	9 (28.1%)	94 (41.4%)
Arms/hands	37 (19.0%)	2 (6.3%)	39 (17.2%)
Legs/feet	38 (19.5%)	11 (34.4%)	49 (21.6%)
Head/neck	35 (17.9%)	10 (31.3%)	45 (19.8%)
**Lesion size**			
< 5 cm	33 (16.9%)	5 (15.6%)	38 (16.7%)
≥ 5 cm	79 (40.5%)	16 (50.0%)	95 (41.9%)
Not documented	83 (42.6%)	11 (34.4%)	94 (41.4%)
**Beam type**			
Electrons	175 (89.7%)	27 (84.4%)	202 (89.0%)
Photons	4 (2.1%)	5 (15.6%)	9 (4.0%)
Not documented	16 (8.2%)	0 (0%)	16 (7.0%)
**Bolus/flab**			
Used	130 (66.7%)	21 (65.6%)	151 (66.5%)
Not used	41 (21.0%)	9 (28.1%)	50 (22.0%)
Not documented	24 (12.3%)	2 (6.3%)	26 (11.5%)
**T-stage (pre-RT)**			
T1–2	35 (17.9%)	11 (34.4%)	46 (20.3%)
T3–4	125 (64.1%)	14 (43.8%)	139 (61.2%)
Not documented	35 (17.9%)	7 (21.9%)	42 (18.5%)
**N-stage (pre-RT)**			
N0	128 (65.6%)	18 (56.3%)	146 (64.3%)
N1–3	11 (5.6%)	1 (3.1%)	12 (5.3%)
Not documented	56 (28.7%)	13 (40.6%)	69 (30.4%)
**M-stage (pre-RT)**			
M0	152 (77.9%)	24 (75.0%)	176 (77.5%)
M1	0 (0%)	1 (3.1%)	1 (0.4%)
Not documented	43 (22.1%)	7 (21.9%)	50 (22.0%)
**Radiotherapy parameters and follow-up**			
RT total dose (Gy), median [Q1; Q3]	8*	34 [30; 38.7]	8 [8; 8]
Dose per fraction (Gy), median [Q1; Q3]	4*	2 [2; 2]	4 [4; 4]
Beam energy (MeV), median [Q1; Q3]	6 [6; 6]	6 [6; 6]	6 [6; 6]
Follow-up duration (months), median [Q1; Q3]	19 [7; 52]	45 [4; 90]	21 [7; 54]

Data are presented as *n* (%) unless otherwise stated; medians are shown with first and third quartiles [Q1; Q3]. Percentages refer to the respective column totals. ‘Not documented’ indicates missing chart documentation. RT: radiotherapy; CTCL: cutaneous T-cell lymphoma.

* All lesions in the low-dose group were treated with two fractions of exactly 4 Gy (total dose 8 Gy); therefore, quartiles are not applicable for these parameters.

The higher-dose group was predominantly delivered with 2 Gy per fraction; therefore, equivalent dose in 2-Gy fractions (EQD2) is effectively equivalent to the physical total dose.

Blood involvement (‘B’) was not analysed due to sparse documentation.

### Follow-up

Follow-up information was obtained from documented clinical follow-up encounters. Endpoint-specific analyses requiring follow-up documentation (in-field relapse and late toxicity) were restricted to lesions with evaluable follow-up. For time-to-event analyses, lesions without the event were censored at the last documented follow-up. Time-to-event outcomes were calculated from the end of RT.

### Outcome

#### Clinical response

Lesion response was categorised according to Olsen et al. using four categories: complete response (CR; no residual lesion), partial response (PR; 50–99% decrease), stable disease (SD; < 50% decrease or < 25% increase) and progressive disease (PD; ≥ 25% increase). OR was defined as CR or PR.

#### In-field relapse and local control

In-field relapse was defined as reappearance or renewed enlargement of a lesion within the irradiated field after an initial CR or PR. Local control time was defined as the time during which a lesion maintained at least a PR after RT; local control rates (LCR) at 1 and 5 years were derived accordingly.

#### Toxicity

Acute and late toxicity signs were extracted from charts and follow-up documentation and graded according to common terminology criteria for adverse events (CTCAE) and/or Radiation Therapy Oncology Group (RTOG)/EORTC as documented in the records [12, 13].

#### Patient satisfaction

Patient satisfaction was assessed in a subsample using the FIPS; the score was calculated as the mean of the six item ratings (range 1 = very satisfied to 6 = very dissatisfied) [14].

### Statistical analysis

Descriptive statistics were used to summarise patient- and lesion-level characteristics. No a priori sample size calculation was performed due to the retrospective design. Statistical significance was set at *p* < 0.05. Response rates (CR, OR) and in-field relapse proportions were compared between dose groups using Fisher’s exact test. Ordinal outcomes (toxicity grades) were compared using the Mann–Whitney U test. Time-to-event endpoints were analysed using the Kaplan–Meier method and compared with log-rank tests. Exploratory analyses of prognostic factors were performed using linear or binary logistic regression models as appropriate; model significance was assessed using chi-square tests. Data were managed in Microsoft Excel (Microsoft Corporation, Redmond, WA, USA), and statistical analyses were conducted in IBM SPSS Statistics (version 29.0; Armonk, NY, USA).

### Ethics

The study was approved by the Ethics Committee of the University of Cologne (application number 21-1045). Procedures involving human participants were conducted in accordance with the ethical standards of the institutional research committee and the 1964 Helsinki Declaration and its later amendments or comparable ethical standards [15].

## Results

### Study cohort and lesion characteristics

Out of 37 screened patient records with primary cutaneous lymphoma treated with focal RT, five patients with cutaneous B-cell lymphoma were excluded. The final cohort comprised 32 patients with CTCL contributing 227 lesions to the lesion-based analysis (195 low-dose; 32 higher-dose). Patients contributed a median of five irradiated lesions per patient (Q25–Q75, 2–10), and some patients had only a single treated lesion. Follow-up was longer in the higher-dose group, reflecting earlier use as the former institutional standard, whereas 2 × 4 Gy was introduced later.

Lesion and treatment characteristics are summarised in Table 1.

### Clinical response

Complete and OR rates were high in both dose concepts (Table 2).

**Table 2:** Key outcomes by dose group (lesion-based cohort).

Outcome	Low-dose RT (2 × 4 Gy)	Higher-dose RT	*P*
**Clinical response (lesion-level)**			
Complete response (CR), *n*/*N* (%)	172/195 (88.2)	29/32 (90.6)	1.000
Overall response (OR = CR + PR), *n*/*N* (%)	184/195 (94.4)	32/32 (100)	0.371
**Local control (time origin = end of RT)**			
Local control rate (LCR) at 1 year, %	82	100	0.031
Local control rate (LCR) at 5 years, %	67	96	0.013
**In-field relapse (restricted to evaluable follow-up)**			
In-field relapse (ever), *n*/*N* (%)	37/184 (20.1)	1/28 (3.6)	-
Time to in-field relapse (months), median [Q1;Q3]	10.7 [7; 22] (*n* = 37)	15.3 (*n* = 1)	-
**Toxicity (CTCAE/RTOG/EORTC; simplified)**			
Acute toxicity at first follow-up: any grade ≥ 1, *n*/*N* (%)	89/195 (45.6)	12/32 (37.5)	0.695
Acute toxicity at first follow-up: grade ≥ 2, *n*/*N* (%)	9/195 (4.6)	4/32 (12.5)	0.695
Late toxicity at last follow-up: any grade ≥ 1, *n*/*N* (%)	33/184 (17.9)	6/28 (21.4)	0.657
Late toxicity at last follow-up: grade ≥ 2, *n*/*N* (%)	5/184 (2.7)	1/28 (3.6)	0.657
Maximum observed toxicity grade	2	2	-
**Patient-reported satisfaction (subsample)**			
Freiburg Index of Patient Satisfaction (FIPS), mean score (1 = very satisfied, 6 = very dissatisfied)	1.27 (*n* = 11 questionnaires)	Not assessed	-

Data are presented as *n*/*N* (%) unless otherwise stated. LCR was calculated from the end date of RT and censored at last documented follow-up. In-field relapse and late toxicity analyses were restricted to lesions with evaluable follow-up (*n* = 184 low-dose; *n* = 28 higher-dose). No toxicity > grade 2 was observed. CTCAE: Common terminology criteria for adverse events; EORTC: European Organisation for Research and Treatment of Cancer; RT: radiotherapy; RTOG: Radiation Therapy Oncology Group.

No statistical comparison was performed for time to relapse due to only one event in the higher-dose group.

Statistical tests: Fisher’s exact test for CR/OR and in-field relapse; log-rank test for LCR; Mann–Whitney U test for toxicity grade distributions (table shows aggregated categories).

### Local control

**Figure 1:**
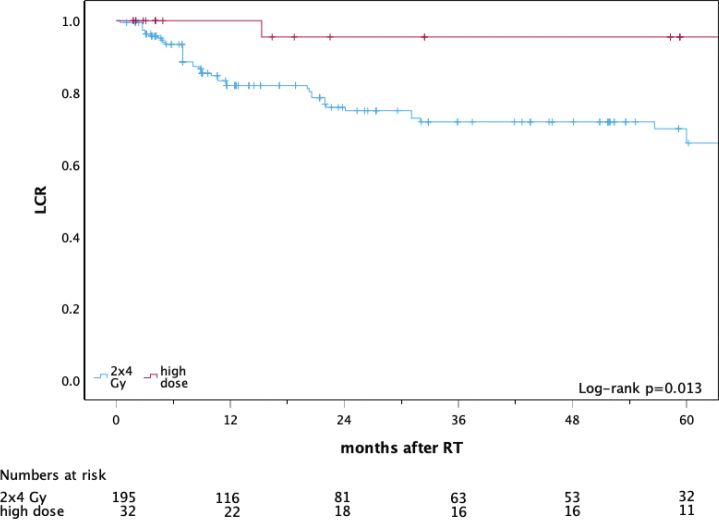
Local control after focal radiotherapy for CTCL lesions: low-dose (2 × 4 Gy) versus higher-dose radiotherapy. Note: Kaplan–Meier curves show local control (LCR; time to loss of local control (in-field relapse)) for irradiated lesions stratified by dose concept: low-dose RT (2 × 4 Gy; n = 195 lesions) and higher-dose RT (n = 32 lesions). Time is shown as months after completion of RT (time zero); lesions were censored at the last documented follow-up (tick marks). Numbers at risk are shown below the x-axis. Between-group differences were assessed using the log-rank test (p = 0.013). CTCL: cutaneous T-cell lymphoma; RT: radiotherapy; LCR: local control rate.

Local control was inferior after low-dose RT (Figure 1, Table 2).

### In-field relapse

Among lesions achieving CR/PR with evaluable follow-up, in-field relapse occurred more frequently after low-dose RT (Table 2). Median time to relapse was 10.7 months (interquartile range [IQR] 7–22).

### Toxicity

Toxicity was low in both groups (Table 2). There were no statistically significant differences in toxicity grades between dose concepts at first follow-up (*p* = 0.695) or last follow-up (*p* = 0.657).

### Patient satisfaction (FIPS)

In a subsample, 11 evaluable FIPS questionnaires were returned; mean FIPS was 1.27 (median 1; 1 = excellent to 6 = very poor), indicating high satisfaction. Between-group comparison was not feasible due to the small subsample and missing responses from patients treated exclusively with higher-dose RT.

### Exploratory analyses (prognostic factors)

Exploratory analyses suggested lower CR rates for SS compared with mycosis fungoides (66.7% vs 89.4%; *p* = 0.019) and for N3 compared with N0 disease (25.0% vs 93.2%; *p* = 0.002). Use of bolus/flab was associated with higher response (OR rate 98% vs 84%; *p* = 0.009) and higher CR (90.7% vs 76%; *p* = 0.001).

## Discussion

This lesion-based retrospective study evaluated focal low-dose RT with 2 × 4 Gy versus higher-dose focal RT (median 34 Gy) for CTCL lesions and indicates a clinically important pattern: initial response rates were excellent and comparable across dose concepts, whereas durability of in-field control favoured higher dosing. This discordance between response rate and durability is highly relevant in CTCL, where focal RT is frequently used for symptom-driven control of selected lesions across disease stages [16–18].

### Response: consistency with prior low-dose focal RT

The high OR rate observed after 2 × 4 Gy is consistent with the established radiosensitivity of CTCL and with published low-dose focal RT series [19, 20]. Neelis et al. reported excellent palliative responses with low-dose schedules and emphasised reduced treatment burden and feasibility of re-irradiation for recurrent lesions [6]. Patel et al. explored dose de-escalation across 4, 8 and 12 Gy in a lesion-based cohort and observed similar high response rates across dose levels [7]. In this context, our data support 2 × 4 Gy as an effective option when the primary clinical objective is prompt lesion reduction and symptom relief rather than durable in-field control.

### Durability of control: dose matters when local control is the goal

In contrast to response, the analyses indicated inferior local control after low-dose RT, with a clear reduction in durability over time. This is compatible with literature suggesting that while response can be achieved at low doses, durability improves with dose escalation [21, 22]. Patel et al. reported superior 1-year local control for 8–12 Gy compared with 4 Gy, indicating a dose–durability relationship even within the low-dose range [7]. Earlier focal RT series in limited-stage mycosis fungoides similarly support that higher doses can translate into robust in-field control [23, 24]. This is echoed in the latest EORTC Cutaneous Lymphoma Tumour Group recommendations, which acknowledge the increasing use of low-dose RT but emphasise the absence of prospective data to standardise doses across CTCL subtypes [5]. In early-stage MF, focal RT is often used for solitary or limited lesions to achieve prolonged remission, while in advanced disease, it serves symptom-driven lesion control. These observations support an intent-based approach: low-dose RT for rapid palliation, and higher-dose RT when durable in-field control is prioritised.

### Toxicity, feasibility and the palliative rationale

Toxicity was low in both groups, with no events beyond grade 2, consistent with the favourable tolerability of focal RT in CTCL. The two-fraction schedule reduces logistic burden and may facilitate repeat focal irradiation if lesions recur, particularly when treatment time and cumulative dose constraints are relevant considerations [25–27]. This rationale aligns with low-dose palliative frameworks in the CTCL RT literature, including single-fraction approaches [6, 28].

Local RT can also be highly effective in selected special clinical presentations, including transformed mycosis fungoides at uncommon sites [29]. Patterns-of-care data suggest that dose de-escalation has not been consistently translated into routine care: Miccio et al. reported that despite recommendations to reduce RT doses below 30 Gy since 2009, only a minority of patients in a large US cohort received low-dose treatment [8]. This underscores the need for clearer intent-based dose selection and dissemination of practical low-dose regimens such as 2 × 4 Gy.

### Exploratory signals: prognostic and technical factors

Exploratory analyses suggested associations between response and clinical/technical variables. Advanced disease characteristics (e.g. SS and advanced nodal status) were associated with reduced CR, and should be interpreted cautiously given subgroup sizes and retrospective documentation. The use of a bolus/flab was associated with higher response rates, potentially reflecting improved superficial dose build-up and target coverage in cutaneous lesions, as discussed for CTCL entities beyond classic MF/SS [30]. Because flab use was not randomised and may correlate with lesion characteristics or physician preference, this finding is hypothesis-generating and supports standardised reporting and prospective evaluation rather than causal inference.

### Strengths and limitations

A key strength of this work is the lesion-based approach aligned with the clinical reality of CTCL, where treatment decisions are frequently driven by individual symptomatic lesions. The cohort includes a substantial number of treated lesions and enables lesion-level assessment of response, local control and in-field relapse.

Limitations primarily reflect the retrospective design and data quality constraints. First, dose selection was non-randomised and likely influenced by clinical context (confounding by indication), while follow-up differed between dose concepts, which can bias long-term comparisons, and the higher-dose group was considerably smaller. Baseline lesion stage also differed between dose concepts (with a higher proportion of tumour-stage lesions in the low-dose group), reflecting potential confounding by indication that may particularly affect durability endpoints. Second, lesion-based analyses may violate independence assumptions because multiple lesions can belong to the same patient; results should therefore be interpreted with awareness of clustering within a patient. Third, reliance on routine documentation led to heterogeneous and incomplete baseline information (including tumor–node–metastasis–blood [TNMB] components, particularly blood involvement, systemic therapy and transformation status such as large cell transformation) and variable follow-up timing, potentially introducing information bias. Fourth, although parts of the project were framed in a non-inferiority logic, the study was not designed as a formal non-inferiority trial and no non-inferiority margin was prespecified; therefore, formal non-inferiority claims should be avoided. Time to response/wound healing and outcomes after re-irradiation (second remission and durability) were not predefined endpoints and could not be assessed reliably from heterogeneous retrospective documentation. Finally, FIPS satisfaction data were obtained only in a subsample and almost exclusively in the low-dose context, limiting comparative conclusions and raising the possibility of investigator bias.

Within-patient comparisons could be valuable in future studies, but were not feasible in our retrospective dataset due to small numbers and heterogeneous lesion characteristics. Prospective, preferably multi-centre studies with standardised assessment schedules are warranted to refine patient- and lesion-level selection. Such studies should use analytic approaches accounting for within-patient lesion clustering and evaluate strategies that may improve durability after low-dose RT.

## Conclusion

Focal low-dose RT with 2 × 4 Gy offers excellent short-term lesion-directed response, low toxicity and minimal treatment burden. Nevertheless, durability of local control was inferior compared with higher-dose focal RT. These findings support positioning 2 × 4 Gy primarily as a short-course regimen optimised for rapid symptom relief and flexibility, whereas higher-dose RT should be favoured when durable in-field control is a central objective with re-irradiation as a pragmatic option.

## Data Availability

All datasets generated or analysed during this study are not publicly available due to institutional and participant privacy considerations but may be made available to qualified researchers upon reasonable request to the corresponding author.
